# A Case of Pill-Induced Esophagitis With Mucosal Dissection

**DOI:** 10.1155/DTE.4.149

**Published:** 1998

**Authors:** Wataru Adachi, Hiroyuki Watanabe, Kazuyuki Yazawa, Naohiko Koide, Shoichiro Koike, Jun Amano, Chisato Tamai

**Affiliations:** 1 Second Department of Surgery Shinshu University School of Medicine 3-1-1 Asahi Matsumoto 390 Japan; 2 Department of Clinical Laboratory Shinshu University School of Medicine 3-1-1 Asahi Matsumoto 390 Japan

## Abstract

With the advance of gastrointestinal endoscopy, pill-induced esophagitis has been detected more frequently, but the association of mucosal dissection is rare. We reported a case of pill-induced esophagitis associated with mucosal dissection.

A 66-year-old male with combined valvular heart disease was admitted for cardiac surgery. Cefotiam hydrochloride tablet was administered for postoperative wound infection of cardiac surgery. Next morning severe odynophagia and retrosternal pain were occurred. Upper gastrointestinal endoscopy performed 2 days after onset of the symptom showed detached mucosa at the upper thoracic esophagus and acute esophagitis at middle and lower thoracic esophagus. Histological examination of the mucosa revealed that the esophageal mucosa was detached from the lamina propria. After the treatment for esophagitis, almost normal esophageal mucosa covered the esophagus without scarring or stricture.

The present case was diagnosed as cefotiam hydrochloride tablet induced esophagitis because of the onset of this disease. Mucosal dissection of the esophagus may be associated with both the esophagitis and bleeding tendencies caused by anticoagulant therapy.


*Diagnostic and Therapeutic Endoscopy*, Vol. 4, pp. 149–153
Reprints available directly from the publisher
Photocopying permitted by license only

© 1998 OPA (Overseas Publishers Association)
Amsterdam B.V. Published under license
under the Harwood Academic Publishers imprint,
part of The Gordon and Breach Publishing Group.
Printed in Singapore.

### A Case of Pill-Induced Esophagitis with Mucosal Dissection

WATARU ADACHI[a,*], HIROYUKI WATANABE[a], KAZUYUKI YAZAWA[a], NAOHIKO KOIDE[a], SHOICHIRO KOIKE[a], JUN AMANO[a] and CHISATO TAMAI[b]

[a] *Second Department of Surgery,* [b] *Department of Clinical Laboratory, Shinshu University School of Medicine, 3-1-1 Asahi, Matsumoto 390, Japan*

*(Received 23 June 1997; Revised 29 July 1997; In final form 25 August 1997)*

**With the advance of gastrointestinal endoscopy, pill-induced esophagitis has been detected more frequently, but the association of mucosal dissection is rare. We reported a case of pill-induced esophagitis associated with mucosal dissection.**

**A 66-year-old male with combined valvular heart disease was admitted for cardiac surgery. Cefotiam hydrochloride tablet was administered for postoperative wound infection of cardiac surgery. Next morning severe odynophagia and retrosternal pain were occurred. Upper gastrointestinal endoscopy performed 2 days after onset of the symptom showed detached mucosa at the upper thoracic esophagus and acute esophagitis at middle and lower thoracic esophagus. Histological examination of the mucosa revealed that the esophageal mucosa was detached from the lamina propria. After the treatment for esophagitis, almost normal esophageal mucosa covered the esophagus without scarring or stricture.**

**The present case was diagnosed as cefotiam hydrochloride tablet induced esophagitis because of the onset of this disease. Mucosal dissection of the esophagus may be associated with both the esophagitis and bleeding tendencies caused by anticoagulant therapy.**

*Keywords:* Pill-induced esophagitis, Mucosal dissection of the esophagus

### INTRODUCTION

Pill-induced esophagitis has not been diagnosed sufficiently, and the true incidence of this disease remains unknown [1]. With the advance of gastrointestinal endoscopy, pill-induced esophagitis should be detected more frequently. Commonly used drugs have been reported as a caustic agent in esophagitis [1–3], and antibiotics are the well-known agents. In contrast, esophageal mucosal dissection, which may be synonymously called submucosal dissection of the esophagus or exfoliative esophagitis, has often been reported due to its characteristic roentgenologic and endoscopic

* Corresponding author. Tel.: 81-263-37-2657. Fax: 81-263-37-2721.

findings [4–8]. In the present report, we present a case of pill-induced esophagitis caused by cefotiam hexetil hydrochloride with esophageal mucosal dissection probably associated with anticoagulant therapy.

### CASE REPORT

A 66-year-old male was admitted to our hospital for cardiac surgery for combined valvular disease; aortic regurgitation (AR), mitral stenosis and regurgitation (MSr), and tricuspid regurgitation (Tr). Chest roentgenography revealed moderate cardiomegaly, and routine echocardiography and transesophageal echocardiography revealed an enlarged left atrium with a small thrombus. There was no history of peptic ulcer or esophageal reflux. Routine upper gastrointestinal endoscopy 2 days before cardiac surgery revealed no abnormal findings including esophagitis, hiatus hernia or peptic ulcer. Aortic and mitral valve replacement, tricuspid annuloplasty and left atrial appendage closure were successfully performed. A thermometer was placed in his esophagus during the operation and a nasogastric tube was placed after the operation for one day. He took a meal from the second postoperative day (POD). After cardiac surgery the patient was given intravenous administration of antibiotics and oral administration of other drugs, such as digoxin, furosemide, spironolactone, varapamil hydrochloride, warfarin potassium, azulene sulfanate sodium · L-glutamine and famotidine. From the 11th POD, an antibiotic (cefotiam hydrochloride) was intravenously administered for leukocytosis. Infection of a femoral wound was noted on the 18th POD, and oral administration of cefotiam hexetil hydrochloride in tablet form (Pansporin T; Takeda Chemical Industries Ltd., Osaka, Japan) from the 19th POD was attempted. Cefotiam hexetil hydrochloride (CHH) is a broad spectrum cephalosporine commonly used in Japan, and its 200 mg tablet measures 13.9 × 8.7 × 7.1 mm. The patient swallowed normally before the 19th POD.

On the 19th POD the patient took the 200 mg CHH tablet with water after his supper. He did not lie down and did not sleep immediately after taking this drug. He felt a slight difficulty in swallowing this tablet because of its large size. The next morning the patient complained of severe odynophagia and retrosternal pain, and could not eat sufficiently. Vomiting or hematemesis was not observed. On the 21st POD a thrombo test showed 12.0% which was low due to the anticoagulant action of warfarin potassium administration.

On the 21st POD a second upper gastrointestinal endoscopy was performed. White detached mucosa was observed on three-quarters of the esophageal circumference and normal mucosa was revealed on the left wall beginning at 25 cm and extending to 30 cm from the incisors (Fig. 1). Reddish esophageal wall was observed deep to the detached mucosa, but hematoma was not observed. The detached mucosa was easily removed, and was examined pathologically. Rough and erosive esophageal mucosa was diffusely observed beginning at 30 cm and extending to 37 cm from the incisors. Slightly erosive gastritis was also observed, but no ulcers were found in the stomach and duodenum. These findings were diagnosed as esophagitis with exfoliation of esophageal mucosa.

Histological examination of the mucosa revealed that the esophageal mucosa was detached from the lamina propria. The basal layer of mucosa was accompanied with many leukocytes. No fungal infection was observed (Fig. 2).

After the second endoscopic examination, administration of CHH tablet was halted and administration of sodium alginate was started. Oral administration of other drugs – digoxin, furosemide, spironolactone, varapamil hydrochloride, warfarin potassium, azulene sulfonate sodium · L-glutamine and famotidine – was continued. The patient returned to normal 2 days after the change in treatment. Follow up endoscopy 18 days after the second endoscopy showed that almost normal esophageal mucosa covered the esophagus without scarring or stricture. The patient was discharged on the 41st POD.

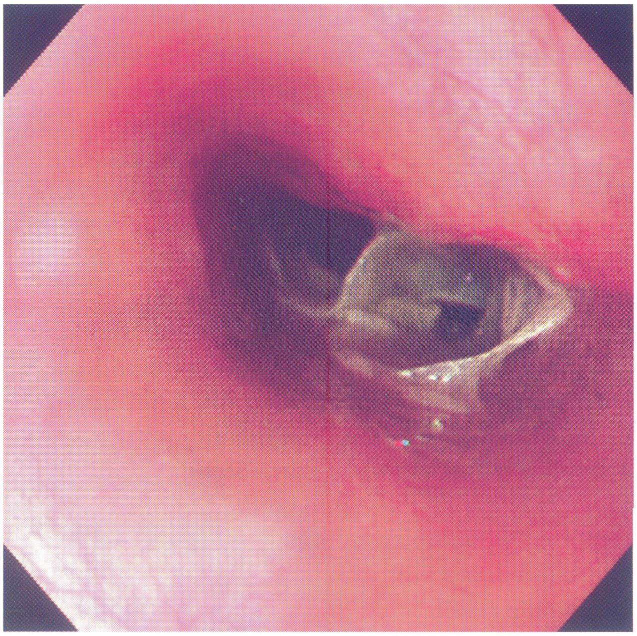

FIGURE 1 Endoscopic photograph taken 24 cm from the incisors. White detached mucosa is revealed.

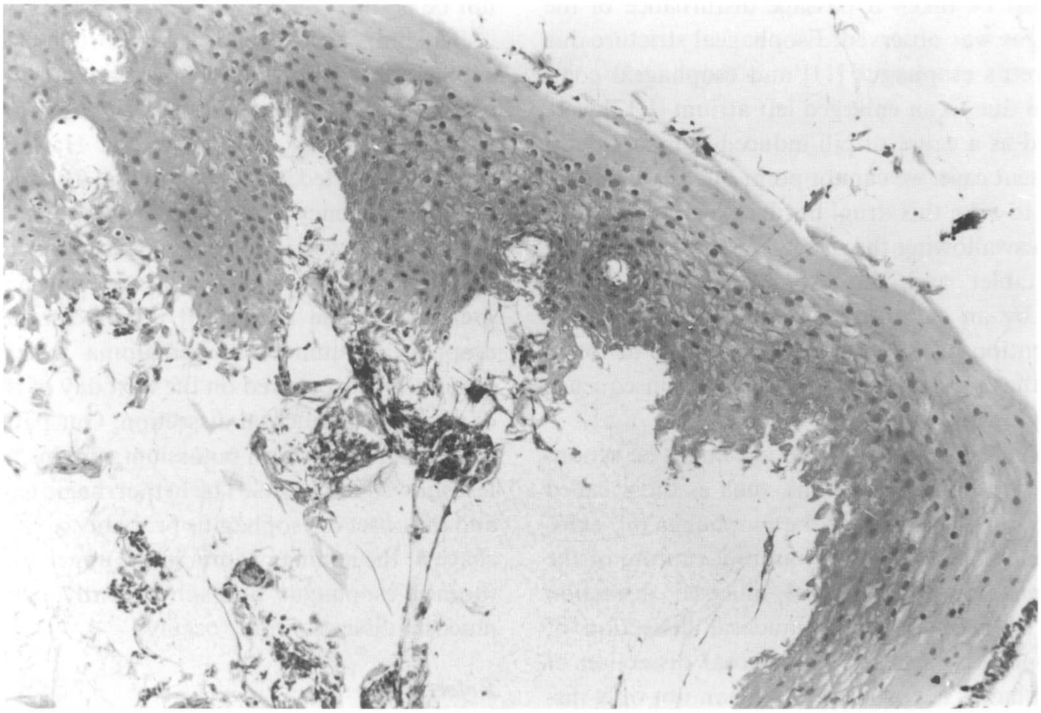

FIGURE 2 Microscope photograph of detached mucosa (HE staining, $\times 100$). Detached mucosa and inflammatory cells are evident.

### DISCUSSION

The present case was diagnosed as CHH-induced esophagitis because the onset of this disease occurred after oral administration of this drug. The patient was not administered other well-known esophagitis-causing agents such as potassium chloride, quinidine, or nonsteroidal antiinflammatory agents. The size of the CHH tablet is large, and a few cases of esophagitis caused by oral administration of the CHH tablet have been reported in Japan. The toxicity of this drug to esophageal mucosa was considered to be its acidity; 200 mg CHH dissolved in 100 ml distilled water showed pH 2.45 [9].

Many drugs have been reported as causing esophageal damage. To prevent pill-induced esophagitis, it is necessary to educate patients in how to take drugs. Taking drugs with plenty of liquid, remaining in an upright position for approximately 1 h after taking medications, and taking drugs at least 1 h before sleeping are important [10]. More care must be taken if passage disturbance of the esophagus was observed. Esophageal stricture due to Barrett's esophagus [11] and esophageal compression due to an enlarged left atrium [1,12] were reported as a cause of pill-induced esophagitis. In the present case, we cannot point out any mistakes in how to take this drug, but he experienced difficulty in swallowing the drug. The large size of the CHH tablet and the esophageal compression caused by an enlarged left atrium may result in the retention of this tablet in the middle or lower part of the thoracic esophagus and as a consequence esophagitis may occur.

Esophageal mucosal dissection might be synonymous with many conditions, such as those called submucosal dissection of the esophagus [6], exfoliative esophagitis [8] or intramural rupture of the esophagus [13]. Esophageal mucosal dissection would be included in submucosal dissection of the esophagus, because submucosal dissection of the esophagus is considered to mean not only dissection at the submucosal layer but also dissection at the layer deeper than the epithelium [4–8]. In our present case, detached mucosa was endoscopically observed, and the mucosa dissected at the lamina propria was histologically revealed. From these findings, the present case was diagnosed as esophageal mucosal dissection.

Drug induced exfoliative esophagitis which was endoscopically characterized by some vertical stripes of thin exfoliative mucosa was reported by Ishii *et al.* [8] and Asada *et al.* [14]. In the present case, however, endoscopic findings were not typical and the mucosal dissection occurred at deeper layer than intraepithelial layer at which drug induced exfoliation frequently occur. The submucosal hemorrhage associated with trauma due to foreign body or vomiting was the most common cause of mucosal dissection [4–7]. In this case, a thermometer and a nasogastric tube were placed prior to the second endoscopy. However, these procedures were not considered to be causes for mucosal dissection, because the procedures were done about 20 days before the mucosal dissection was observed. Other mechanical stimuli to the esophagus could not be pointed out.

Benjamin and Hanks [6] reported a case of submucosal dissection of the esophagus due to spontaneous intramural hemorrhage following administration of anticoagulants. Harada *et al.* [15] also reported a case of mucosal dissection with bleeding tendency. Although esophageal hematoma was not observed in the present case, we could not completely deny that the hematoma had been present. Harada *et al.* [15] reported a case with esophageal submucosal hematoma which endoscopically disappeared on the next day of the onset of esophageal mucosal dissection. Our patient was administered warfarin potassium, and his thrombo test showed only 12%. The hemorrhagic tendencies and the onset of esophagitis probably cause hemorrhage at the lamina propria in the upper part of the thoracic esophagus, and subsequently, esophageal mucosal dissection may occur.

### *References*

[1] Eng, J. and Sabanathan, S. Drug-induced esophagitis. *Am. J. Gastroenterol.* 1991; **86**: 1127–1133.

[2] Bott, S., Prakash, C. and McCallum, R.W. Medication-induced esophageal injury: survey of the literature. *Am. J. Gastroenterol.* 1987; **82**: 758–763.

[3] Lewis, J.H. Gastrointestinal injury due to medicinal agents. *Am. J. Gastroenterol.* 1986; **81**: 819–834.

[4] Tamai, O., Okusima, N., Tomori, T. *et al.* A case of esophageal mucosal dissection during chemotherapy for breast cancer. *Gastroenterol. Endosc.* 1994; **36**: 351–355 (in Japanese).

[5] Kamiya, Y., Ozeki, N., Tanaka, A. *et al.* Report of two cases of esophageal submucosal dissection. *Gastroenterol. Endosc.* 1987; **29**: 2037–2043 (in Japanese).

[6] Benjamin, B. and Hanks, T.J. Submucosal dissection of the oesophagus due to haemorrhage: a new radiographic finding. *J. Laryngol.* 1965; **79**: 1032–1038.

[7] Joffe, N. and Millan, V.G. Postemetic dissecting intramural hematoma of the esophagus. *Radiology* 1970; **95**: 379–380.

[8] Ishii, K., Mitsuhashi, T., Imaizumi, H. *et al.* Endoscopic study on esophageal ulcers of the upper and middle esophagus. *Gastroenterol. Endosc.* 1992; **34**: 363–371 (in Japanese).

[9] Maeda, K., Amatsu, T., Masaki, H. *et al.* A case of esophageal ulcer caused by Pansporin T tablet. *Gastroenterol. Endosc.* 1992; **34**: 709 (in Japanese).

[10] Goldberg, N.E., Ott, M.J. and Hartigan, L.P. Medication-induced esophagitis. *J. Pediatr. Health Care* 1996; **10**: 35–36.

[11] Chami, T.N., Nikoomanesh, P. and Katz, P.O. An unusual presentation of pill-induced esophagitis. *Gastrointest. Endosc.* 1995; **42**: 263–265.

[12] Pemberton, J. Oesophageal obstruction and ulceration caused by oral potassium therapy. *Br. Heart. J.* 1970; **32**: 267–268.

[13] Marks, I.N. and Keet, A.D. Intramural rupture of the oesophagus. *Br. Med. J.* 1968; **3**: 536–537.

[14] Asada, S., Nabeshima, T., Miyashi, H. *et al.* Three cases of exfoliative esophagitis. *Gastroenterol. Endosc.* 1985; **27**: 578 (in Japanese).

[15] Harada, H., Tomoda, J., Uesaka, K. *et al.* A case of esophageal mucosal denudation in patient receiving hemodialysis. *Gastroenterol. Endosc.* 1987; **29**: 538–542 (in Japanese).

